# Microbiological, pathological and histological findings in four Danish pig herds affected by a new neonatal diarrhoea syndrome

**DOI:** 10.1186/1746-6148-9-206

**Published:** 2013-10-12

**Authors:** Hanne Kongsted, Beata Jonach, Svend Haugegaard, Øystein Angen, Sven E Jorsal, Branko Kokotovic, Lars E Larsen, Tim K Jensen, Jens P Nielsen

**Affiliations:** 1 Pig Research Centre, Danish Agriculture & Food Council, Vinkelvej 13, Kjellerup 8620, Denmark; 2 National Veterinary Institute, Technical University of Denmark, Bülowsvej 27, Frederiksberg C 1870, Denmark; 3 HERD – Centre for Herd-oriented Education, Research and Development, Department of Large Animal Sciences, University of Copenhagen, Groennegaardsvej 2, Frederiksberg C 1870, Denmark

## Abstract

**Background:**

Neonatal diarrhoea is a frequent clinical condition in commercial swine herds, previously regarded to be uncomplicated to treat. However, since 2008 it seems that a new neonatal diarrhoeic syndrome unresponsive to antibiotics and common management practices has emerged. Routine laboratory examinations have not detected any pathogen related to this syndrome. The primary purpose of this study was to evaluate if well-known enteric pathogens could be associated with outbreaks of neonatal diarrhoea, thus question the hypotheses of a new syndrome. Furthermore, we wanted to evaluate macroscopic and microscopic findings associated with these outbreaks and if possible propose a preliminary piglet-level case-definition on syndrome New Neonatal Porcine Diarrhoea syndrome (NNPDS).

**Results:**

Four well-managed herds experiencing neonatal diarrhoea with no previously established laboratory conclusion and suspected to suffer from New Neonatal Porcine Diarrhoea Syndrome, were selected. Within these herds, 51 diarrhoeic and 50 non-diarrhoeic piglets at the age of three to seven days were necropsied and subjected to histological and microbiological examination. Faeces were non-haemorrhagic. Neither enterotoxigenic *E. coli*, *Clostridium perfringens* type A or C, *Clostridium difficile*, rotavirus, coronavirus, *Cryptosporidium spp*, *Giardia spp*, *Cystoisospora suis* nor *Strongyloides ransomi* were associated with diarrhoea in the investigated outbreaks. Macroscopically, the diarrhoeic piglets were characterized by filled stomachs and flaccid intestines without mucosal changes. The predominant histological lesions were villous atrophy in jejunum and ileum. Epithelial lesions in colon were seen in one third of the case piglets.

**Conclusions:**

The results of the study supported the hypothesis that a new neonatal porcine diarrhoea was present in the investigated herds, since no known pathogen(s) or management factors could explain the diarrhoeal outbreaks. Based on the findings in the four herds the following case-definition of NNPDS was suggested: Non-haemorrhagic diarrhoea during the first week of life, without detection of known infectious pathogens, characterized by milk-filled stomachs and flaccid intestines at necropsy.

## Background

Neonatal diarrhoea is a well-known clinical condition, present at varying prevalence in most commercial swine herds. However, since 2008 field experiences on an apparently new diarrhoeic syndrome unresponsive to antibiotics and common management practices have been reported (personal communications, S.E. Jorsal, National Veterinary Institute, Technical University of Denmark and B. Svensmark, Pig Research Centre, Danish Agriculture & Food Council, Denmark). The emergence of a new neonatal diarrhoeic syndrome (by some authors referred to as New Neonatal Porcine Diarrhoea (NNPD) has been suggested in different countries [1-4]. A common feature of the reported cases is that known enteric pathogens cannot be associated with the clinical outbreaks in routine laboratory submissions.

Routine laboratory testing protocols may vary from region to region. Disregarding local procedures, the following agents are usually included in diagnostic protocols for neonatal diarrhoea: Enterotoxigenic *Escherichia coli* (ETEC), *Clostridium perfringens* type A (CPA), *Clostridium perfringens* type C (CPC), *Clostridium difficile* (CD) rotavirus group A (RV) and coronavirus [1,5]. Parasites, which may be relevant to consider in relation to neonatal diarrhoea are *Cryptosporidium spp*, *Giardia spp*, *Cystoisospora suis* and *Strongyloides ransomi*[6-8]. Systematic investigations of piglets from herds affected by the apparently new diarrhoeic syndrome are lacking.

The overall aim of this study was to investigate whether a detailed microbiological examination of a larger number of piglets from affected herds could link the presence of neonatal diarrhoea with known enteric pathogens. Such associations would challenge the hypothesis that a new disease syndrome has evolved. Another aim was to determine if diarrhoeic piglets from different herds had characteristic and consistent gross and microscopic lesions to support the elaboration of a joint case definition of NNPDS.

The article describes the prevalence of well-known enteric pathogens in age-matched diarrhoeic- and non-diarrhoeic piglets from four herds affected by neonatal diarrhoea with no previously established laboratory conclusion. Furthermore, results of gross pathology and histopathology are presented. Summarizing these findings, the article suggests a case-definition on NNPDS.

## Results

### Epidemiologic data on piglets

A total of 51 diarrhoeic (11–14 pr. herd) and 50 non-diarrhoeic piglets (12–13 pr. herd) at the age of three to seven days were included in the study. Clinically, the diarrhoeas were non-haemorrhagic. Eighty percent of diarrhoeic piglets had been diarrhoeic for either two or three days prior to euthanasia. Diarrhoea for four days was seen in 14% of the diarrhoeic piglets whereas only 6% had been diarrhoeic for five days.

### Microbiology

Table 1 summarizes the microbiological findings in relation to diarrhoeic status. None of the microbiological agents was significantly more prevalent in diarrhoeic than in non-diarrhoeic piglets.

**Table 1:** Microbiological findings in diarrhoeic and non-diarrhoeic piglets

**Microbiological agent**	**Diarrhoeic**	**Non-diarrhoeic**	***P-value***^**1**^
	**n=51**	**n=50**	
	**(%)**	**(%)**	
Haemolytic *E. coli* non-typable	6	0	0.1
Non-haemolytic *E. coli* non-typable	47	48	0.5
Non-haemolytic *E. coli* O-rough	4	2	0.5
Non-haemolytic *E. coli* serogroup O8	4	6	0.8
Non-haemolytic *E. coli* O157	8	0	0.06
*C. perfringens* type A	35	70	0.9
*C. perfringens* type C	6	2	0.3
*C. difficile*	0	4	0.5
rotavirus group A	2	0	0.5
coronavirus	0	0	1

^1^: Positive associations with diarrhoea tested by One-sided Fisher’s exact test.

Non-haemolytic *E. coli* was the predominant finding in the aerobic culture from both diarrhoeic and non-diarrhoeic piglets, whereas haemolytic strains were found in only three piglets in total (all of them diarrhoeic). The main part of *E. coli* isolates were non-typeable. Sixty-three *E. coli* isolates were subjected to virulence gene determination by PCR. Fimbrial genes were detected in nine of 35 isolates from diarrhoeic piglets. The fimbrial distribution among isolates was; F4 (n=2), F5 (n=1), F6 (n=1), F18 (n=1), F41 (n=2), F5/F6 (n=1) and F5/F41 (n=1). In non-diarrhoeic piglets fimbrial genes were detected in seven of 28 isolates. The fimbrial distribution among these isolates was; F4 (n=1), F5 (n=1), F6 (n=1), F18 (n=2) and F41 (n=2). Table 2 gives an overview of toxin genes detected in fimbriated and non-fimbriated isolates from the two groups of piglets. Classic ETEC with simultaneous occurrence of both fimbrial and toxin genes were detected in only one diarrhoeic piglet.

**Table 2:** **Occurence of toxin genes within fimbriated and non-fimbriated *E. coli *isolates from diarrhoeic and non-diarrhoeic piglets**

***E. coli *isolates**	**n**	**STa**^**1**^	**STb**^**1**^	**LT**^**2**^	**VT2e**^**3**^	**STa/LT**	**STb/VT2e**	**LT/VT2e**
**From diarrhoeic piglets**								
Fimbriated	9	1	0	0	0	0	0	0
Non-fimbriated	26	1	2	2	5	0	0	0
**From non-diarrhoeic piglets**								
Fimbriated	7	0	0	0	3	0	0	0
Non-fimbriated	21	1	0	0	3	1	1	1

^1^: Heat-stable enterotoxins a and b. ^2^: Heat-labile enterotoxin. ^3^: Verotoxin 2e.

In the anaerobic culture, CPA was a very frequent finding. These bacteria were more prevalent in non-diarrhoeic than in diarrhoeic piglets (70% vs. 35%).

### Necropsy

Necropsy findings are presented in Table 3. Very few extra-intestinal lesions were observed (not shown), and only one of these; a pale or icteric liver, was observed in more than one piglet (5 of the diarrhoeic vs. 1 of the non-diarrhoeic piglets).

**Table 3:** Necropsy findings in diarrhoeic and non-diarrhoeic piglets

**Necropsy findings**	**Diarrhoeic**	**Non-diarrhoeic**	***P-value***^***1***^
	**n=51**	**n=50**	
	**(%)**	**(%)**	
*General findings*			
**Poor body condition**	**57**	**4**	**< 0.0001**
**Dehydration**	**29**	**2**	**< 0.0001**
Empty stomach	0	12	1
*Small intestine*			
**Flaccidity**	**73**	**20**	**< 0.0001**
Hyperaemia of serosa	6	0	0.2
Striping of serosa	2	0	1
Edema in mesentery	4	4	1
Enlargement of lymph nodes	18	16	0.9
Dullness/ necrosis of mucosa	8	8	1
**Watery contents**	**57**	**30**	**0.01**
*Large intestine*			
**Flaccidity**	**53**	**6**	**< 0.0001**
Edema in mesentery	39	20	0.06
Enlargement of lymphnodes	4	2	1
**Liquid contents**	**48**	**10**	**< 0.0001**

^1^: One-sided Fisher’s exact test. Findings having a statistically significant positive association with diarrhoea are presented in bold.

**Figure 1:**
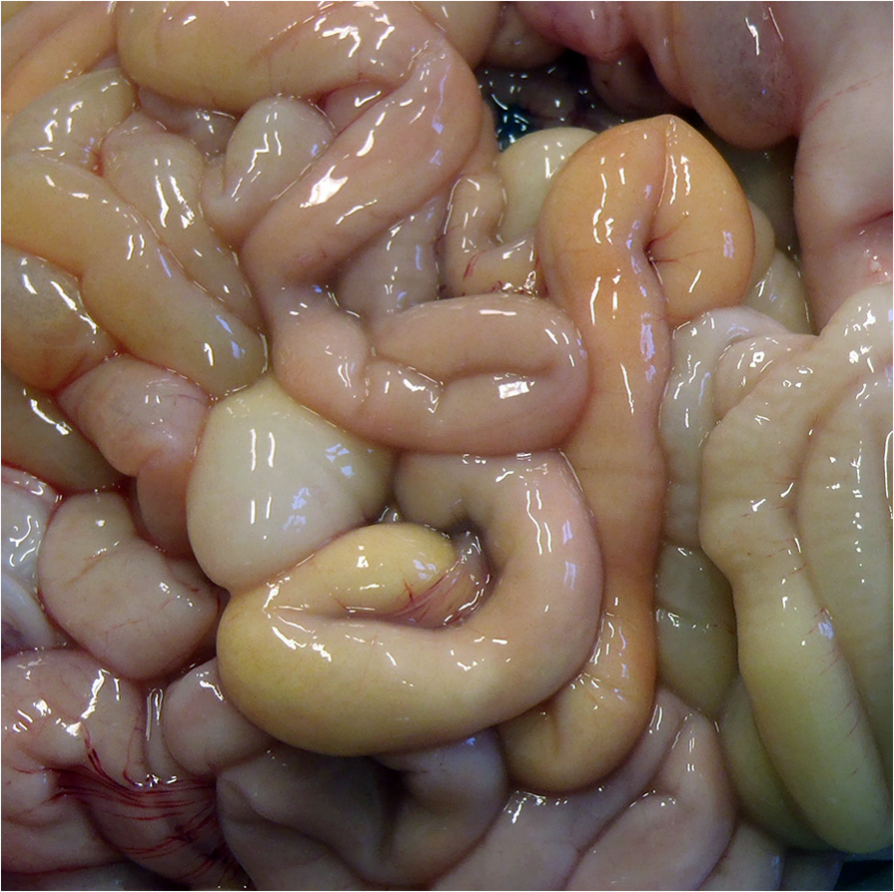
**Flaccid intestine of 3 days old diarrhoeic piglet.** The small intestine is thin-walled and flaccid throughout its length. The intestine appears to lack its normal peristaltic capacity, since no sections are contracted. Colon (in the right side of the picture) has liquid contents.

**Figure 2:**
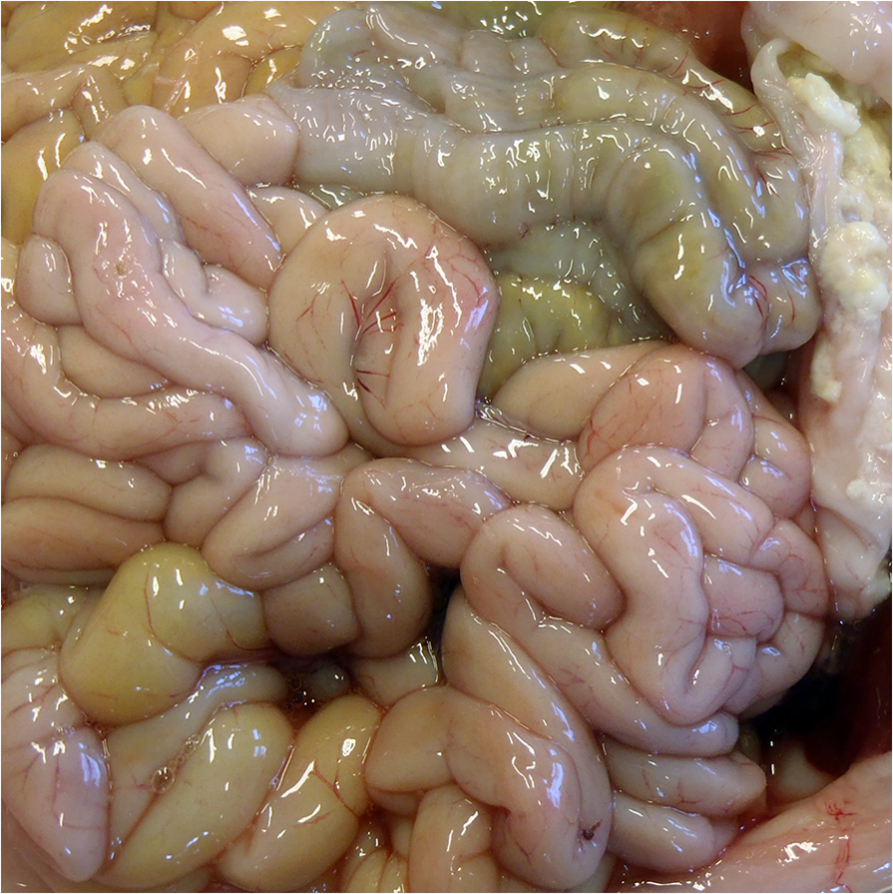
**Normal intestine of 3 days old non-diarrhoeic piglet.** The small intestinal wall has a normal thickness. Different parts of the intestine show different stages of peristalsis, reflecting normal peristaltic capacity.

As indicated in Table 3, six findings showed a statistically significant higher prevalence in diarrhoeic versus non-diarrhoeic piglets. Table 4 outlines the prevalence of these six findings in the two groups of piglets within each herd. Within all herds, a poor body condition, flaccidity of the small intestine, flaccidity of the large intestine and liquid large intestinal contents seemed positively associated with diarrhoea (though not statistically significant in all cases, see Table 4). Flaccidity of the large intestine was in most cases (25 of 27 cases) seen in conjunction with small intestinal flaccidity. Figure 1 shows a flaccid and Figure 2 shows a normal intestine.

**Table 4:** Necropsy findings in diarrhoeic- (D) and non-diarrhoeic (ND) piglets in individual herds

**Necropsy findings**	**Herd 1**		**Herd 2**		**Herd 3**		**Herd 4**	
	**D**	**ND**	**D**	**ND**	**D**	**ND**	**D**	**ND**
	**n=13**	**n=13**	**n=11**	**n=12**	**n=14**	**n=13**	**n=13**	**n=12**
	**(%)**	**(%)**	**(%)**	**(%)**	**(%)**	**(%)**	**(%)**	**(%)**
*General findings*								
Poor body condition	77*	15	63*	0	57*	0	31*	0
Dehydration	31*	0	0	0	57*	8	23	0
*Small intestine*								
Flaccidity	69*	15	81*	25	86*	0	54	42
Watery contents	92*	23	64*	17	36	31	38	50
*Large intestine*								
Flaccidity	62*	8	55*	8	64*	0	31	8
Liquid contents	46*	8	45	17	50*	8	46*	8

* denotes significant statistical positive association with diarrhoea (one-sided Fisher’s exact test (α=0.05)) within the individual herds.

### Histopathology

**Figure 3:**
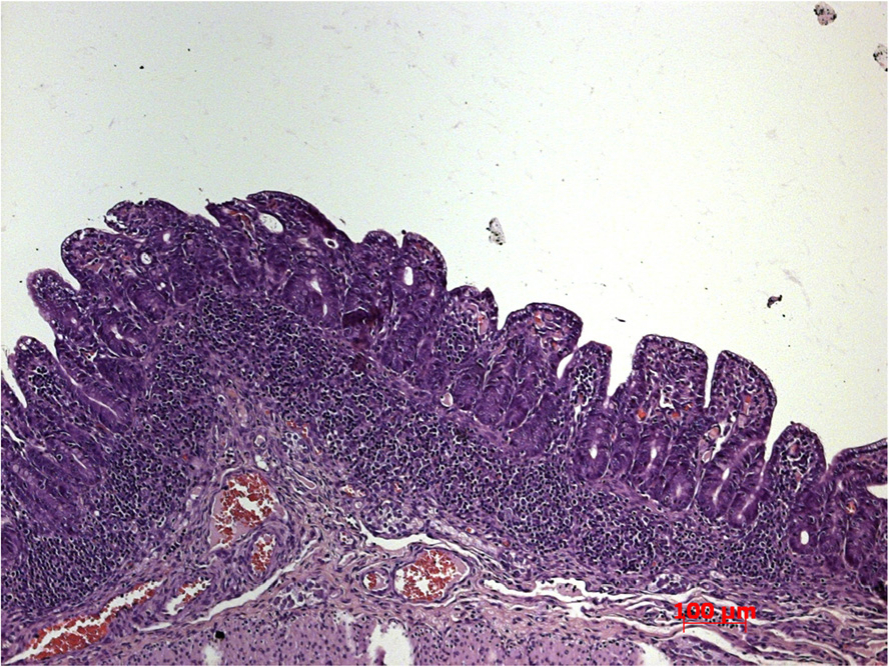
Severe villous atrophy in ileum of 5 days old diarrhoeic piglet.

**Figure 4:**
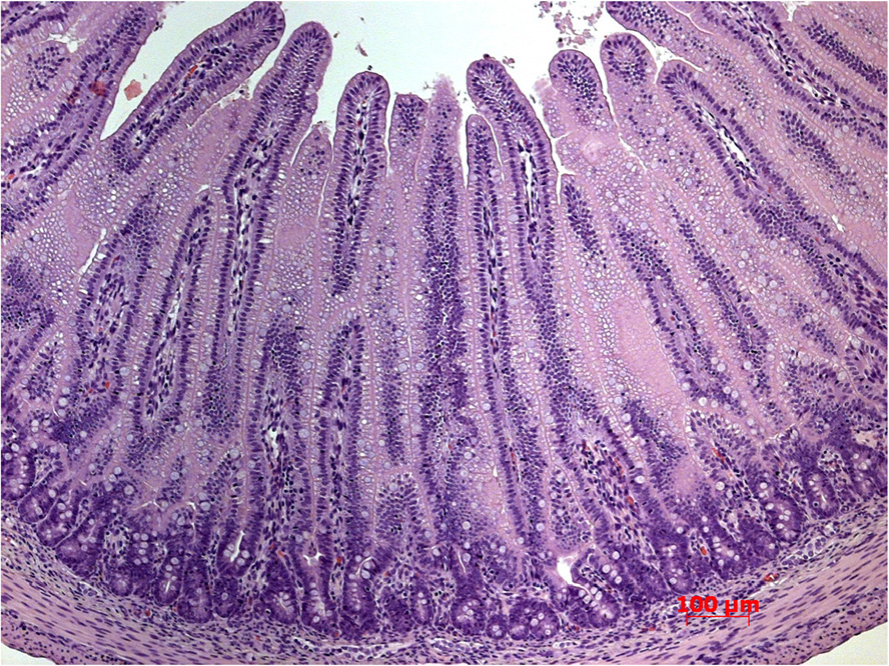
Normal intestinal villi in ileum of 5 days old non-diarrhoeic piglet.

In the small intestine, villous atrophy with crypt hyperplasia was the most frequently observed lesion. Figure 3 shows atrophic villi in ileum as compared to normal villi shown in Figure 4. Overall, an atrophic pattern was seen in the jejunal and/or ileal mucosa in 63% of diarrhoeic and 12% of non-diarrhoeic piglets. The severity of atrophy varied, with no obvious association with diarrhoeic status. In ileum, the villous atrophy was most pronounced over the Peyer’s patches. Duodenal villi were not affected. A statistically significant association (P<0.001) between villous atrophy and flaccidity of the small intestine at necropsy was seen. In 76% of piglets having villous atrophy, small intestinal flaccidity had been recorded at necropsy.

Irrespective of diarrhoeic status approximately 30% of piglets had a slight to moderate local infiltration of neutrophils in the lamina propria. Occasionally, the lamina propria in the diarrhoeic piglets was congested and edematous.

Mild epithelial lesions were seen at the tip of the villi in 20% of the diarrhoeic and 6% of the non-diarrhoeic piglets and were usually associated with villous atrophy. Crypts of Lieberkühn epithelium were intact in both groups. Foci of mucosal necrosis were seen in the small intestines of 6% of the diarrhoeic piglets versus none of the non-diarrhoeic ones. In colon, mild epithelial lesions were seen in 33% of the diarrhoeic piglets and 11% of the non-diarrhoeic piglets. Occasionally, the colonic crypts in diarrhoeic piglets were irregular and elongated. Mucosal necrosis in the colon was seen in one diarrhoeic piglet, which also had necrotic changes in the small intestine.

No parasites were seen in the intestinal mucosa of any piglet.

Table 5 depicts histopathological findings in diarrhoeic and non-diarrhoeic piglets within the four herds and summarizes the overall prevalences. Both villous atrophy and large intestinal epithelial lesions showed an overall statistically significant positive association with diarrhoea, and seemed positively (or at least not negatively) associated with diarrhoea within all herds.

**Table 5:** Main histological findings in 51 diarrhoeic (D) and 50 non-diarrhoeic (ND) piglets

**Histological findings**	**Herd 1**		**Herd 2**		**Herd 3**		**Herd 4**		**In total**	
	**D**	**ND**	**D**	**ND**	**D**	**ND**	**D**	**ND**	**D**	**ND**
	**n=13**	**n=13**	**n=11**	**n=12**	**n=14**	**n=13**	**n=13**	**n=12**	**n=51**	**n=50**
	**(%)**	**(%)**	**(%)**	**(%)**	**(%)**	**(%)**	**(%)**	**(%)**	**(%)**	**(%)**
*Small intestine*										
**Villous atrophy**	62*	8	82*	8	71*	0	38	33	**63**	**12**
Neutrophil infiltration	38	38	55	25	21	15	23	50	33	32
**Epithelial lesions**	15	15	36*	0	21	0	8	8	**20**	**6**
Mucosal necrosis	8	0	0	0	0	0	15	0	6	0
*Large intestine*^1^										
**Epithelial lesions**	17	15	55*	8	46	22	15	0	**33**	**11**
Mucosal necrosis	0	0	0	0	0	0	8	0	2	0

^1^: In 2 diarrhoeic and 4 non-diarrhoeic piglets samples from colon were missing or autolytic and therefore not included in the analysis.

*denotes statistically significant positive associations with diarrhoea within herds (one-sided Fisher’s exact test (α=0,05)). Findings having a statisticallly significant association with diarrhoea across herds are presented in bold.

## Discussion

Non-enterotoxigenic (containing neither fimbrial nor toxin genes) *E. coli* was a frequent finding in both diarrhoeic and non-diarrhoeic piglets, whereas only one enterotoxigenic isolate was detected. Hence, ETEC did not seem to play any pathogenic role in relation to the investigated outbreaks of diarrhoea. Other studies have indicated that attaching and effacing *E. coli* (AEEC), carrying neither fimbrial nor toxin genes, are able to induce diarrhoea in newborn piglets [9] and to induce villous atrophy [10]. The prevalence of AEEC in the present study is currently being investigated.

CPC was cultured in four piglets of the study. Due to the low prevalence the significance of this bacterium in relation to the investigated outbreaks is probably minimal. The significance of CPA in relation to diarrhoea in neonatal piglets is controversial, since it has been concomitantly recognized as part of the normal intestinal flora and as a potential intestinal pathogen [11,12]. In this study we found a significantly higher prevalence of CPA in non-diarrhoeic vs. diarrhoeic piglets. Most likely, this finding merely reflects the intact intestinal flora within the non-diarrhoeic piglets. CD has been reported in cases of neonatal diarrhoea in piglets [5,12]. However, in this study, this bacterium was only detected in two piglets and the characteristic histopathological lesions previously reported to be associated with CD infections [13] were not seen. Accordingly, CD does not seem to be associated with the investigated outbreaks.

The scarcity of known pathogens in the outbreaks of the study supports the hypothesis that the investigated herds experienced diarrhoea of unknown aetiology. Therefore the outbreaks may be representative of the new syndrome NNPDS.

A poor body condition and dehydration were rather prevalent findings in diarrhoeic piglets in this study. However, unless very pronounced, these features are not characteristic for specific diarrhoeic syndromes, since they merely reflect the loss of nutrients and water associated with any diarrhoeic condition. Milk-filled stomachs, in contrast, seem to be a characteristic finding associated with this syndrome. Since neonatal diarrhoea is commonly associated with malabsorption caused by starvation, the filled stomachs seen in 100% of diarrhoeic piglets in this study are interesting findings which clearly differentiate this syndrome from outbreaks of neonatal diarrhoea related to starvation. However, since the vast majority (80%) of piglets in this study were diarrhoeic for two or three days only, we do not have information on the contents of stomachs at later stages of disease. Obviously, one would expect long lasting diarrhoea to keep piglets from suckling due to malaise, and therefore this criterion is probably only valid at early stages of disease.

Intestinal flaccidity was the most prominent and consistent gross lesion. Flaccidity of intestines is seen in different conditions, Postweaning Multisystemic Wasting Syndrome (PMWS) [14] and diet-induced malabsorption being the most obvious examples. Liquid contents in colon are expected in all diarrhoeic conditions and are therefore not considered diagnostic to any specific syndrome. Somewhat surprising, half of the diarrhoeic piglets did not have liquid content in colon at necropsy, which probably reflects that these piglets were in the recovery phase of disease. If so, this potentially implies a diagnostic problem due to less pronounced lesions and decreased excretion of infectious agents at this phase. However, since the clinical course of diarrhoea turned out to be very short (as evidenced by 80% of the selected piglets being diarrhoeic for only two or three days) it would not have been practically feasible to avoid selection of piglets in recovery.

The most consistent and predominant histological lesion observed in diarrhoeic piglets was villous atrophy (seen in 63% of diarrhoeic vs. 12% of non-diarrhoeal piglets). Villous atrophy is a very common finding in diarrhoeic conditions [15] and in this study, the atrophy was neither associated with infection by well-known pathogens nor malnutrition. The strong association between villous atrophy and grossly visible intestinal flaccidity indicates that decreased mucosal thickness is reflected grossly as a thin-walled, atonic intestine. Epithelial lesions in the large intestine also seemed to be consistently associated with diarrhoea in this study (seen in 33% of diarrhoeic vs. 11% of non-diarrhoeic piglets), but due to the low prevalence, these lesions do not seem to be relevant to include in a case definition.

Overall, the present study suffers from lack of comparable piglets from non-NNPDS-affected herds in order to correctly classify findings as typical or diagnostic of NNPDS. Moreover, the selection of study herds posed some difficulties since the selection was basically based on a high prevalence of diarrhoea and absence of agents (in five piglets). Obviously, potential misclassification of herds is an issue to consider – though hard to address or control at this stage of investigation.

To our knowledge, this is the first study investigating outbreaks of diarrhoea in herds suspected to suffer from NNPDS. The aetiology behind these outbreaks was either undetected pathogens or non-infectious factors. Practical experience indicates that eg. high levels of protein in sow feed can lead to diarrhoea in neonatal pigs. However, all of the investigated herds used restricted levels of protein in sow feed, and had previously tried minimizing protein content with no preventive effect. As previously underlined, the diarrhoea seemed unrelated to postnatal starvation, bot intrauterine events may have affected the normal development of intestinal absorptive capacity. Thus, the villous atrophy seen in the study may reflect prenatal under-development of villi.

The unspecific nature of intestinal lesions seen in this study underlines the complexity of intestinal pathology in neonatal pigs. Interestingly, even early studies from the seventies and eighties concluded that gross lesions seem similar and unspecific irrespective of the underlying aetiology in this age group of piglets [16,17]. Moreover, in the study from 1975 no pathogens were detected in as many as 32% of fatal neonatal gastroenteropathies. Thus, the existence of neonatal diarrhoa with unspecific lesions and without known pathogens is not a new phenomenon. It appears, however, that the clinical picture in the herds is new. At this point we do not know whether this clinical picture is a more severe manifestation of a syndrome already present but not recognized back in the seventies, or if we are experiencing a truly new syndrome.

From a practical point of view, obviously the most urgent issue is to recognize the aetiology behind the current problems. This study disclaims associations with established agents, but yet unestablished agents or shifts in intestinal bacteral population dynamics may play a role. Therefore, culture-independent methods like metagenomics and high-throughput qPCR may be rewarding in the future investigation.

## Conclusions

This study suggests the existence of a yet unexplained diarrhoea syndrome related to the first week of life. The syndrome is not related to starvation or infection by enterotoxigenic *E. coli*, *Clostridium perfringens* type A or C, *Clostridium difficile*, rotavirus, coronavirus, *Cryptosporidium spp*, *Giardia spp*, *Cystoisospora suis* or *Strongyloides ransomi*. Characteristic postmortem findings are flaccid intestines without mucosal pathology or lymph node enlargement. Histologically, villous atrophy in jejunum and ileum are the most prominent findings and epithelial lesions in colon also seem to be associated with the syndrome. Apparently, the flaccidity of intestines seen at necropsy is a reflection of the reduced length of intestinal villi. For a preliminary piglet level case-definition we suggest the following; Non-haemorrhagic diarrhoea during the first week of life, with no detection of known infectious agents and characterized by a milk-filled stomach and flaccid intestines at necropsy. Since histopathological examination mainly revealed uncharacteristic lesions, this diagnostic approach does not seem to be rewarding at this stage of investigation.

## Methods

### Study design

A case–control study on 101 euthanized piglets selected from four Danish production herds was performed during 2011. A total of 989 piglets from these herds were clinically evaluated from the day of birth and 110 were euthanized at selected time points.

### Inclusion of herds

Herds were recommended by veterinary practitioners and included in accordance with the following criteria: 1) Presence of diarrhoea responding poorly to antibiotics during the first week of life (at least 30% affected litters for a period of minimum 6 months), 2) Routine vaccination of sows against ETEC and CPC, 3) Failure of preventive management interventions, 4) PRRS negative farrowing unit as demonstrated in blood samples tested by ELISA/IPT or PCR and 5) Negative results of routine diagnostic examinations for ETEC, CPC and RV in five diarrhoeic piglets aged one to four days.

A total of four herds were selected. They all presented high standards of housing and management with all- in/ all-out practice in farrowing units and appropriate cleaning between farrowing batches. Farrowing crates had partially slatted floors of plastic or iron bars with supplemental heat and cover provided for the piglets.

All four herds had been affected by neonatal diarrhoea for at least one year. Preventive interventions which had failed included minimizing protein-levels in sow feed, optimization of hygiene procedures, immunization by faecal backfeeding and vaccination against CPA and Porcine circovirus type 2 (PCV2). In all herds, many different antibiotics and different treatment strategies had been tried out unsuccessfully. All herds used Toltrazuril at day three to four of life to prevent coccidiosis. Castration of males and iron-injections were carried out at the same day. Descriptive data on the herds are presented in Table 6.

**Table 6:** Descriptive data of the 4 herds in the study

**Herd data**	**Herd 1**	**Herd 2**	**Herd 3**	**Herd 4**
Study period	January 2011	March 2011	May 2011	July 2011
Herd size (number of sows)	900	1250	700	950
SPF^1^-status	Not declared	Not declared	SPF+AP12	SPF
Piglets weaned/sow/year^2^	30.7	27.1	25.4	32.3
1st parity litters (%)^2^	20	22	21	23
Recruitment of gilts	Purchase	Own production	Purchase	Own production
Sow feed^3^	Wet/ Home made	Wet/ Home made	Wet/ Home made	Wet/ Factory made

^1^: Specific Pathogen Free - disease surveillance programme. Danish herds can participate in this programme which registers presence of certain infectious diseases, including PRRS, *M. hyopneumoniae, A. pleuropneumoniae, P. multocida* tox+ and *B. hyodysenteriae.*^2^: Average values calculated from herd-registrations made in a 3-month-period prior to investigation. ^3^: Feed type used in farrowing period.

### Inclusion of sows and piglets

In each herd, approximately 20 newly farrowed sows (half of a farrowing batch) with no clinical signs of disease prior to farrowing were selected. The selection procedure was designed to include all available first parity litters, since they were expected to exhibit the highest prevalence of diarrhoea. At the day of birth (day one), the included litters were standardized to 11 or 12 piglets by randomly selecting piglets weighing ≥ 800 g. Surplus piglets were removed during the first 16 hours after birth and no cross-fostering was made during the suckling period. Sows and piglets were clinically examined daily from day 1 until day five to seven and again on day ten. In the same period, rectal swabs were taken. Consistency of faeces was judged as fluid or normal from the appearance on the rectal swab.

### Definition on cases and controls and selection procedure

In each herd, age-matched case and control piglets were selected for necropsy at two different time-points – early and late in the course of disease. These time-points were based on previous experience with the syndrome in each herd. In herds experiencing diarrhoea starting at the second day of life, piglets were necropsied at day three and five of life. If diarrhoea occurred from the third day of life, piglets were necropsied at day four and six of life and so on. In the 4 herds, clinical signs started at day two, three or four of life, resulting in necropsies being performed on piglets between three and seven days of age.

Selection criterion for diarrhoeic piglets was fluid consistency of faeces for at least two subsequent days, including the day of selection. Selection criterion for non-diarrhoeic piglets was normal consistency of faeces at all days prior to selection. Diarrhoeic piglets were selected from the litters having the highest prevalence of diarrhoea, whereas non-diarrhoeic piglets were selected from the litters exhibiting no or little diarrhoea. None of the piglets euthanized at the early stage (three to five days of age, depending on herd) had been treated by antibiotics. All diarrhoeic piglets euthanized at the late stage (five to seven days of age, depending on herd) had been medicated according to the individual herd routine.

### Diagnostic procedures

#### Necropsy

Live piglets were transported to the laboratory and euthanized within six hours after selection. All organs were routinely examined for gross lesions. A poor body condition was recorded if protruding ribs and spine were observed. Dehydration was recorded if eye balls were deeply positioned in the skull and muscles appeared dry on the cut surface.

#### Histopathology

Histopathological examination was carried out on samples from duodenum, jejunum, ileum and spiral colon. Samples were fixed immediately after euthanasia in 10% neutral buffered formalin for at least 48 hours. The samples were then embedded in paraffin wax, cut at 3 μm, stained with haematoxylin and eosin (HE) and examined by light microscopy. The intestinal mucosa of each specimen was histopathologically evaluated.

Villous atrophy was recorded when shortening of villi accompanied by decreased height of enterocytes and increased cellularity of the lamina propria was seen in at least one region of the intestinal sample. Crypt hyperplasia was recorded when the intestinal crypts were elongated with an increased number of mitotic figures. Disruptions in normal epithelial architecture with preserved integrity of the epithelium were recorded as mild epithelial lesions. Diffuse necrotic changes in the epithelium and lamina propria were recorded as mucosal necrosis.

The presence of parasites (*Cryptosporidium spp*, *Giardia spp*, *Cystoisospora suis* and *Strongyloides ransomi*) was examined using standard diagnostic criteria.

#### Bacteriology

Sections of jejunum and colon were aerobically cultured for *E. coli*. Parallel culturing on Drigalski (in house selective and indicative medium for coliforms) and blood agar plates (Columbia agar (Oxoid) supplemented with 5% calf blood) was performed. Plates were incubated for 24 hours at 37°C. Piglets were considered *E. coli* positive if any growth of haemolytic colonies or moderate/ massive growth of non-haemolytic colonies was seen in any section of intestine. Serogrouping of *E. coli* was performed - using one isolate per piglet - by agglutination with monovalent O-antisera (O8, O45, O64, O138, O139, O141, O149 and O157, Statens Serum Institut, Copenhagen, Denmark) [18] and real-time PCR was performed for detection of virulence factor genes F4, F5, F6, F18, F41, STa, STb, LT and VT2e [18]. If no agglutination with antisera was seen, the isolate was designated non-typeable. If agglutination occurred in all pools, the isolate was considered to be O-rough.

Culturing of CP was carried out using Columbia agar (Oxoid) supplemented with 5% calf blood and polymyxin incubated anaerobically for 24 hours at 37°C. Colonies were verified using Tryptose-Sulfite-Cycloserine agar (Oxoid). Piglets were considered culture positive if moderate/ massive growth was observed in any section of intestine. Typing of suspected samples was performed by PCR [19] on a pool of four isolates having characteristic colony morphology on Columbia agar (shiny, grey, double-haemolytic colonies). Culturing of CD was performed using Cycloserine Cefoxitin Fructose Agar, incubated anaerobically for 48 hours at 37°C. Piglets were considered positive if yellow colonies with a characteristic horse-stable odour were detected in any section of intestine.

#### Virology

Contents of jejunum were examined for rotavirus group A by an enzyme immunoassay (ProSpectT® Rotavirus) according to the manufacturer’s instructions and for coronavirus by a pan-corona RT-PCR assay as previously described [20].

#### Statistics

Positive associations between diarrhoea and microbiological and pathological findings were evaluated using one-sided Fisher’s exact tests (α=0.05). When considered relevant, associations between histopathology and necropsy were also evaluated by Fisher’s exact tests (α=0.05). Since the piglets originated from four different herds, associations within herds were also assessed.

### Ethical approval

The present study was not subject to ethical approval as Danish laws do not require ethical approval for studies not involving different treatment groups or blood testing. The study only involved procedures normally used for routine diagnostics.

## Competing interests

The authors declare that they have no competing interests.

## Authors’ contributions

All authors contributed to the design of the study. Inclusion of herds, clinical examination in the herds and selection of piglets was done by HK. Necropsy was performed by SH and histological examinations were performed by BJ. Culturing of *Clostridium difficile* was done by BK and virulence gene determination of *E. coli* isolates by ØA. LEL did the pan-corona RT-PCR assays. HK conducted the statistical analysis. All authors participated in drafting the manuscript and proofreading of the final manuscript.
